# Predictors of Severity and Mortality in Chronic Liver Disease Patients With COVID-19 During the Second Wave of the Pandemic in India

**DOI:** 10.7759/cureus.20891

**Published:** 2022-01-03

**Authors:** Pankaj Nawghare, Shubham Jain, Sanjay Chandnani, Saurabh Bansal, Sameet Patel, Partha Debnath, Siddhesh Rane, Rahul Deshmukh, Pravin Rathi, Qais Contractor

**Affiliations:** 1 Gastroenterology, BYL Nair Hospital, Mumbai, IND

**Keywords:** curb-65, acute on chronic liver failure, d-dimer, covid-19, cirrhosis

## Abstract

Background

Coronavirus disease 2019 (COVID-19) infection in chronic liver disease patients is associated with poor outcomes. In this study, we aimed to evaluate the predictors of severity and mortality in this group of patients during the second wave of the COVID-19 pandemic in India. In addition, we compared cirrhotic patients with COVID-19 with cirrhotic patients from the pre-COVID-19 period.

Methodology

This was a single-center observational study. We included data from 50 patients with cirrhosis and COVID-19 retrospectively from the discharge/death files. A comparison group of 100 patients with cirrhosis from the pre-COVID period was also analyzed retrospectively.

Results

The majority of patients had predominantly respiratory symptoms, with fever being the most common symptom (85%). The most common presentation was acute on chronic liver failure (ACLF). The most common form of decompensation was jaundice followed by hepatic encephalopathy. The overall mortality in cirrhotic patients with COVID-19 was double than that in cirrhotic patients from the pre-COVID-19 period. All patients with ACLF succumbed to multiorgan failure. Diabetes was the only comorbidity that was associated with severe infection. Higher creatinine on admission and high D-dimer levels correlated with severity. D-dimer was the only parameter that correlated with severity and mortality on multivariate analysis. None of the comorbidities predicted mortality. Among various composite scores, the Child-Turcotte-Pugh (CTP) score and CURB-65 correlated with mortality. On the area under the receiver operating characteristic analysis, a D-dimer level of >1.1 mg/L was associated with mortality.

Conclusions

COVID-19 infection in patients with cirrhosis is associated with poor outcomes. D-dimer levels of >1.1 mg/L on admission are a simple parameter to predict mortality. CTP and CURB-65 are composite scores that correlate with mortality in this group of patients.

## Introduction

In December 2019, patients with unexplained pneumonia were discovered in Wuhan, Hubei Province, China, which was caused by a novel coronavirus [1]. The coronavirus disease 2019 (COVID-19) then rapidly spread to the rest of the world including India. After a declining trend of cases during the start of 2021, there was a rapid surge in COVID-19 cases from February 2021 in India, which led to the second wave of the pandemic. Recent data suggest that at present the mutant virus is much more infectious than it was in the first wave; however, the number of daily deaths is lower [2].

Although COVID‐19 is primarily a respiratory illness [3], it affects other organs such as the heart, liver, pancreas, and kidneys [4]. The liver can be involved because of the ubiquitous distribution of viral entry receptors, namely, angiotensin-converting enzyme 2 (ACE2). COVID‐19‐associated liver injury is defined as any liver damage occurring during disease progression and treatment of COVID‐19 in patients with or without any pre‐existing liver disease [4]. The overall incidence of elevated serum liver biochemistries in patients with COVID‐19 ranged from 14% to 53% during the first wave of the pandemic worldwide [5]. Elevation in aspartate transaminase (AST) and alanine transaminase (ALT) is usually mild (i.e., less than five times the upper limit of normal). According to previous studies reported during the first wave, low albumin is a marker of severe infection and poor prognosis [6].

Cirrhosis represents a major disease burden globally. Given this high burden, the impact of underlying liver conditions on the outcome in COVID-19 patients needs to be evaluated. Several studies were conducted during the first wave of COVID-19 to assess its impact on chronic liver disease (CLD) patients. Hence, we conducted this study to determine the outcomes of the patients and to assess the predictors of severity and mortality in patients with cirrhosis with COVID-19 (CWC) infection during the second wave.

## Materials and methods

Study design and data collection

This single-center, observational study was conducted at a tertiary care center. We recruited 50 CWC patients admitted from February 2021 to June 2021. Patients were diagnosed as COVID-19 (reverse transcription polymerase chain reaction/rapid antigen positive for SARS-CoV-2) based on the Indian Council of Medical Research (ICMR) criteria [7]. Patients were categorized and managed as per the ICMR guidelines [8]. They were also categorized based on symptoms into predominantly respiratory symptoms group, predominantly gastrointestinal symptoms group, and both respiratory and gastrointestinal symptoms group. Data for all patients were collected retrospectively from discharge cards/death summaries. Liver-related complications were managed as per standard guidelines [9]. Acute on chronic liver failure (ACLF) was defined as “an acute deterioration of pre-existing CLD usually related to a precipitating event and associated with increased mortality at three months due to multisystem organ failure” as per the European Association of the Study of Liver-Chronic Liver Failure (EASL-CLIF) consortium definition. Grading of ACLF was done as per the EASL-CLIF-C grading. We collected cirrhosis without COVID-19 (CWOC) patient data retrospectively from the discharge cards from the pre-COVID-19 period and matched (age and gender) with the study group.

Inclusion and exclusion criteria

We included COVID-19 patients aged >18 years with CLD diagnosed based on the clinical, laboratory, and radiological findings. We excluded pregnant women from this study.

Ethical clearance

The study protocol was approved by the Institutional Ethics Committee before the commencement of the study (approval number: ECARP/2020/88).

Statistical analysis

Normally distributed data were expressed as mean ± standard deviation (SD). Nominal data were expressed as frequency and percentage. The Student’s t-test was used for comparing continuous data as appropriate. The chi-square test or Fisher’s exact test was used for categorical variables whenever applicable. Univariate and multivariate Cox regression analyses were performed to determine the predictors of in-hospital mortality. A p-value of <0.05 was considered significant. Data were analyzed using SPSS statistical software, version 22 (IBM Corp., Armonk, NY, USA).

## Results

In this study, we analyzed a total of 150 patients (50 CWC patients and 100 CWOC patients).

Cirrhosis with COVID-19 (study group)

Age and Sex Distribution

The mean age of the study population was 49 years. The majority of patients were male, with a male-to-female ratio of 2.125:1.

Clinical Presentation

Most patients (21/50) presented with predominantly respiratory symptoms, with fever being the most common symptom (85%), followed by cough (60%). Predominantly gastrointestinal symptoms were seen in 24% of patients (12/50). Among gastrointestinal symptoms, the most common symptom was jaundice (83%). The remaining 17 (34%) patients had both respiratory and gastrointestinal symptoms on presentation. Among 50 CWC patients, the most common presentation was ACLF (40%), followed by acute decompensation (AD) (34%). COVID-19 severity was mild in 13 patients, moderate in nine patients, and severe in 28 patients. Mortality was 0%, 11.11% (1/9), and 85.7% (24/28) in the mild, moderate, and severe groups, respectively.

Patients with compensated cirrhosis (n = 13): The majority of patients (11/13) presented with respiratory symptoms. Of the other two patients, one had diarrhea and the other had abdominal pain. COVID-19 severity was mild in 10 patients and moderate in three patients. All three patients with moderate severity required oxygen supplementation in the form of nasal cannula. None of the patients required invasive ventilation. All patients with compensated cirrhosis were discharged. The mean hospital stay was 9 ± 3.89 days for these patients.

Patients with AD (n = 17): The majority of patients (11/17) had involvement of both respiratory and gastrointestinal systems. The most common form of decompensation was jaundice (14/17), followed by hepatic encephalopathy (5/17). Of the 17 patients, eight (47%) had severe COVID-19 infection followed by moderate infection in six (35%) patients. All three patients with mild infection were maintaining oxygen saturation and were discharged. Among patients with moderate severity, four required nasal oxygen supplementation, and one patient required bag and mask ventilation (BMV). The remaining one patient required invasive ventilation and succumbed to the illness. All patients in the severe category required oxygen supplementation: BMV in four patients, non-invasive ventilation (NIV) in one, and invasive ventilation in three patients. All patients on invasive ventilation and NIV died. The mean hospital stay was 13.65 ± 7.42 days in these patients.

Patients with ACLF (n = 20): Of the 20 patients who had ACLF, 11 had ACLF on admission, and nine developed ACLF during the hospital stay. Among them, 65% presented with grade III ACLF. The cause of decompensation was alcohol intake in seven patients, hepatotoxic drugs in four, and superimposed viral infection in three patients. However, in six patients, the cause of decompensation could not be identified. All patients with ACLF had severe COVID-19 infection. All patients required oxygen supplementation: invasive ventilation in 13, NIV in four, and BMV in three patients. All patients succumbed due to multiorgan failure. The mean hospital stay was 8 ± 4.29 days in these patients.

Treatment

Of the various antibiotics, azithromycin was most commonly used (54%). Ivermectin and remdesivir were administered in 42% and 36% of patients, respectively. Although CWC patients were started on heparin without prior endoscopy, none of them had gastrointestinal bleeding post-treatment.

Table 1 presents the characteristics of CWC patients.

**Table 1:** Characteristics of CWC patients. CWC: cirrhosis with COVID-19; CLD: chronic liver disease; ALD: alcoholic liver disease; HBV: hepatitis B virus; NASH: non-alcoholic steatohepatitis; AIH: autoimmune hepatitis; HCV: hepatitis C virus; ACLF: acute on chronic liver failure; AD: acute decompensation; HTN: hypertension; DM: diabetes mellitus; CVD: cardiovascular disease; COPD: chronic obstructive pulmonary disease; CKD: chronic kidney disease; MELD: Model for End-Stage Liver Disease

Parameters	Number of patients (n = 50) (%)
Age (years), mean (SD)	49.1 (10.9)
Age group (years)	
<40	10 (20.0)
≥40 to <60	29 (58.0)
≥60	11 (22.0)
Sex	
Male	36 (72.0)
Female	14 (28.0)
Cause of CLD	
ALD	24 (48.0)
HBV	13 (26.0)
NASH	5 (10.0)
AIH	4 (8.0)
HCV	2 (4.0)
Cryptogenic	2 (4.0)
Symptoms	
Respiratory	21 (42.0)
Gastrointestinal	12 (24.0)
Gastrointestinal and respiratory	18 (36.0)
Liver disease	
ACLF	20 (40.0)
Acute decompensation	17 (34.0)
Compensated	13 (26.0)
ACLF grade (n = 20)	
1	0 (0)
2	7 (35.0)
3	13 (65.0)
Type of AD (n = 17)	
Encephalopathy	6 (35.3)
Jaundice	5 (29.4)
Ascites	4 (23.5)
Bleeding	2 (11.8)
Comorbidities	
HTN	19 (38.0)
DM	11 (22.0)
CVD	7 (14.0)
COPD	2 (4.0)
CKD	3 (6.0)
No comorbidity	16 (32.0)
MELD score, median (range)	21.5 (6.0-49.0)
MELD Na score, median (range)	21.5 (6.0-49.0)
CURB-65 score	
0-1	16 (32.0)
2	15 (30.0)
3-5	19 (38.0)
Child-Turcotte-Pugh score, median (range)	9.0 (5.0-15.0)
Treatments	
Heparin	33 (66.0)
Steroid	28 (56.0)
Azithromycin	27 (54.0)
Ivermectin	21 (42.0)
Remdesivir	18 (36.0)
Oxygen therapy	
Mechanical ventilation	22 (44.0)
Nasal cannula	6 (12.0)
Bag and mask	3 (6.0)
Non-invasive ventilation	3 (6.0)
Duration of hospitalization (days) (mean ± SD)	11.1 ± 6.2

Comparison between severe and non-severe subgroups of the cirrhosis with COVID-19 group

Among 50 CWC patients, 28 had severe COVID-19 infection. Mortality in CWC with severe infection was more than the non-severe subgroup (85.7% vs 11.11%; p < 0.05). On univariate analysis, three parameters were associated with severity. Diabetes was the only comorbidity associated with severe infection (odds ratio [OR] = 0.106, 95% confidence interval [CI] = 0.244-0.012; p = 0.032). Among various laboratory parameters, high creatinine at admission was associated with severe disease (OR = 0.228, 95% CI = 0.079-0.008; p < 0.05). Raised D-dimer was also associated with severe infection (OR = 0.379, 95% CI = 0.005-0.027; p = 0.006). However, other inflammatory markers such as C-reactive protein and serum ferritin were not significant. On multivariate analysis, D-dimer (OR = 1.145, 95% CI = 1.011-1.298; p = 0.034) was the only parameter that was associated with severe disease (Table 2).

**Table 2:** Covariates associated with severity. OR: odds ratio; CI: confidence interval; MELD: Model for End-Stage Liver Disease; HTN: hypertension; DM: diabetes mellitus; COPD: chronic obstructive pulmonary disease; CKD: chronic kidney disease; SGOT: serum glutamic oxaloacetic transaminase; INR: international normalized ratio; NLR: neutrophil-to-lymphocyte ratio; CRP: C-reactive protein

Parameters	Univariate analysis			Multivariate analysis		
	OR	95% CI	P-value	OR	95% CI	P-value
Age	0.069	-0.002, 0.008	0.214	-	-	-
Sex	0.040	-0.073, 0.163	0.439	-	-	-
MELD score	0.087	-0.010, 0.016	0.642	-	-	-
Child-Turcotte-Pugh score	0.312	0.011, 0.20	0.35	-	-	-
HTN	-0.044	-0.158, 0.068	0.422	-	-	-
DM	0.106	0.244, 0.012	0.032	0.452	0.081, 2.517	0.365
COPD	-0.018	-0.304, 0.212	0.719	-	-	-
CKD	-0.093	-0.427, 0.036	0.094	-	-	-
Hemoglobin (g/dL)	-0.030	-0.046, 0.033	0.734	-	-	-
Platelet count (10^3^/µL)	0.063	-0.001, 0.004	0.376	-	-	-
Total bilirubin (mg/dL)	0.177	-0.006, 0.023	0.225	-	-	-
SGOT (U/L)	0.041	-0.001, 0.002	0.720	-	-	-
Total protein (g/dL)	0.039	-0.100, 0.175	0.579	-	-	-
Albumin (g/dL)	-0.070	-0.353, 0.251	0.732	-	-	-
Creatinine (mg/dL)	0.228	0.079, 0.008	0.018	0.944	0.607, 1.467	0.797
INR	0.111	-0.010, 0.046	0.193	-	-	-
NLR	-0.112	-0.046, 0.019	0.402	-	-	-
D-dimer (mg/L)	0.379	0.005, 0.027	0.006	1.145	1.011, 1.298	0.034
CRP (mg/L)	0.106	-0.002, 0.005	0.273	-	-	-
Ferritin (ng/mL)	-0.141	-0.004, 0.000	0.106	-	-	-

Comparison between survival and non-survival subgroups of the cirrhosis with COVID-19 group

**Figure 1:**
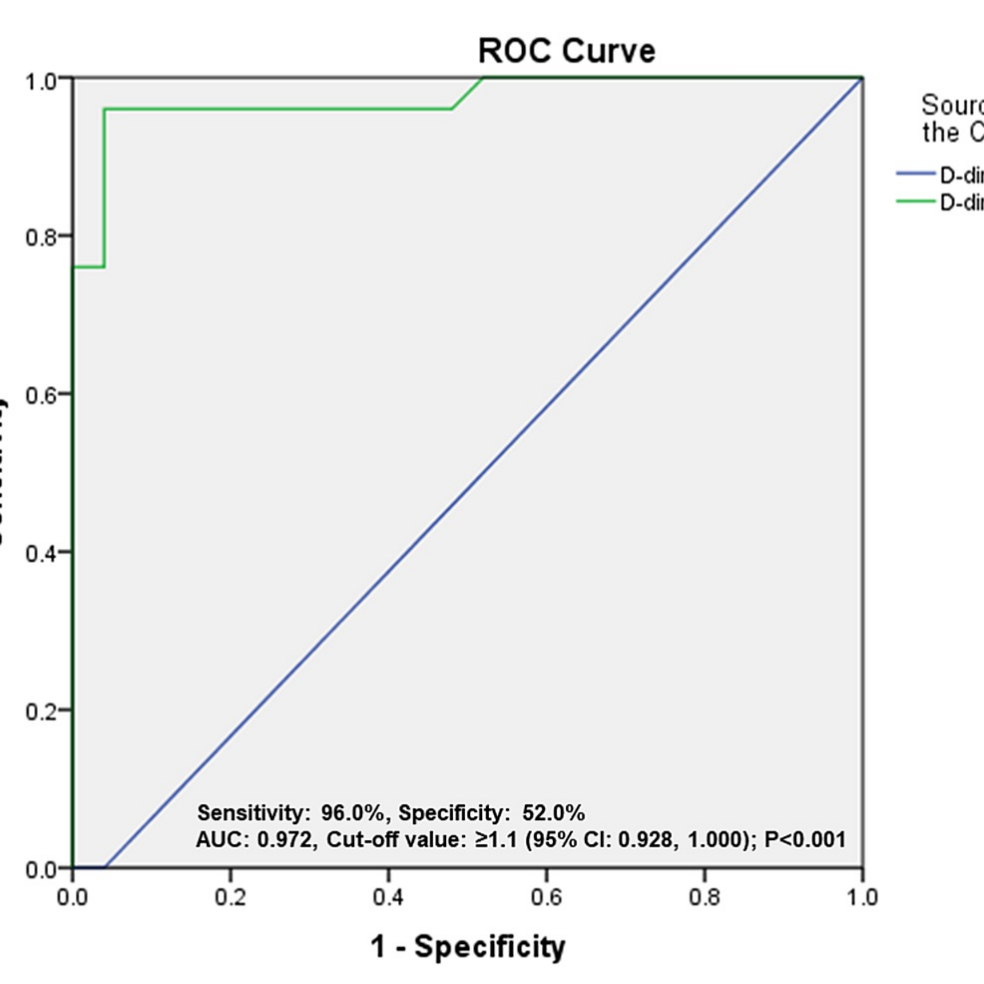
ROC curve of D-dimer levels. ROC: receiver operating characteristic

Overall mortality in CWC patients was 50%. On univariate analysis, the CURB-65 and Child-Turcotte-Pugh (CTP) scores on admission correlated with mortality (p < 0.05). None of the comorbidities such as diabetes and chronic kidney disease were associated with mortality. Among laboratory parameters, D-dimer and lymphocyte count were associated with mortality (p < 0.05). The CURB-65 score, CTP score, lymphocyte count, and D-dimer were included in the multivariate logistic regression for mortality in CWC patients, which showed that only raised D-dimer (OR = 1.374, 95% CI = 1.004-1.880; p = 0.047) was associated with mortality. On the area under the receiver operating characteristic (AUROC) curve, D-dimer of >1.1 g/L could predict mortality with a sensitivity of 96% and specificity of 52%, with an area under the curve (AUC) of 0.972 (p < 0.001) (Figure 1).

Comparison between cirrhosis with COVID-19 and the historical control group

Additionally, we compared CWC with CWOC patients from the pre-COVID-19 period. Historical controls were matched for both age and sex. Mortality was double in CWC patients which was statistically significant (50% vs. 25%, p = 0.003). The most common presentation in CWC patients was ACLF (40% vs. 20%, p = 0.001). However, in the historical cohort, most patients presented with AD (34% vs. 63%, p = 0.001). Among CWC patients, the most common type of decompensation was hepatic encephalopathy (35.3% vs. 16.4%, p = 0.001). All patients in the CWC group had a higher grade of ACLF in contrast to the historical control group (grade III ACLF 65% vs. 45%, p = 0.001). Among various laboratory parameters, liver enzymes, creatinine, INR, Model for End-Stage Liver Disease (MELD) score, and MELD-Na score were significantly higher compared to the historical cohort (Tables 3, 4).

**Table 3:** Comparison between CWC and CWOC cohorts. CWC: cirrhosis with COVID-19; CWOC: cirrhosis without COVID-19; COVID-19: coronavirus disease 2019; CLD: chronic liver disease; HBV: hepatitis B virus; NASH: non-alcoholic steatohepatitis; AIH: autoimmune hepatitis; HCV: hepatitis C virus; EHPVO: extrahepatic portal venous obstruction; BCS: Budd-Chiari syndrome; AD: acute decompensation; ACLF: acute on chronic liver failure; TLC: total leucocyte count; SGOT: serum glutamic oxaloacetic transaminase; SGPT: serum glutamic pyruvic transaminase; INR: international normalized ratio; CTP: Child-Turcotte-Pugh; MELD: Model for End-Stage Liver Disease

Parameter	COVID-19-positive (n = 50)	Historical controls, cirrhosis patients (n = 100)	P-value
Age (years)			
<60	36 (72.0)	89 (89.0)	0.011
≥60	14 (28.0)	11 (11.0)	
Sex			
Male	34 (68.0)	72 (72.0)	0.704
Female	16 (32.0)	28 (28.0)	
Etiology of CLD			
Alcoholic liver disease	24 (48.0)	33 (33.0)	0.260
HBV	13 (26.0)	23 (23.0)	
NASH	5 (10.0)	9 (9.0)	
AIH	4 (8.0)	7 (7.0)	
Cryptogenic	2 (4.0)	9 (9.0)	
HCV	2 (4.0)	6 (6.0)	
Wilson’s disease	-	8 (8.0)	
EHPVO	-	3 (3.0)	
BCS	-	2 (2.0)	
Clinical presentation			
AD	17 (34.0)	67 (67.0)	0.001^*^
ACLF	20 (40.0)	20 (20.0)	
Type of AD			
Ascites	4 (23.5)	28 (41.8)	0.001^*^
Bleeding	2 (11.8)	13 (19.4)	
Jaundice	5 (29.4)	15 (22.4)	
Hepatic encephalopathy	6 (35.3)	11 (16.4)	
ACLF grade			
I	-	6 (30)	0.001^*^
II	7 (35)	5 (25)	
III	13 (65)	9 (45)	
Laboratory parameter, median (range)			
Hemoglobin (g/dL)	8.7 (4.5-12.4)	8.6 (4.8-13.4)	0.298
Platelet (10^3^/µL)	101.0 (24.0-145.0)	97.5 (19.0-214.0)	0.908
TLC (10^3^/µL)	8.9 (3.3-39.2)	7.2 (2.5-16.0)	<0.001
Total bilirubin (mg/dL)	5.4 (0.3-34.2)	2.3 (0.5-32.5)	0.061
SGOT (U/L)	78.0 (23.0-256.0)	61.5 (0.9-456.0)	0.004^*^
SGPT (U/L)	53.5 (14.0-187.0)	37.0 (13.0-273.0)	0.019^*^
Total protein (g/dL), mean (SD)	6.4 (0.5)	6.3 (0.7)	0.264
Albumin (g/dL)	2.8 (1.5-3.8)	2.9 (1.5-6.3)	0.316
Alkaline phosphate (U/L)	168.0 (72.0-369.0)	144.5 (2.9-363.0)	0.022^*^
Creatinine (mg/dL)	1.4 (0.5-13.4)	1.0 (0.5-4.1)	0.006^*^
INR	1.8 (0.8-11.4)	1.1 (0.8-5.8)	<0.001^*^
CTP	9.0 (5.0-15.0)	8.0 (5.0-14.0)	0.144
MELD	23.5 (6.0-40.0)	13.0 (6.0-40.0)	0.003^*^
MELD-Na	21.5 (6.0-49.0)	16.0 (6.0-40.0)	0.018^*^
Sodium (mmol/L)	134.0 (116.0-145.0)	135.5 (125.0-147.0)	0.778
Outcome			
Discharge	25 (50.0)	75 (75.0)	0.003^*^

**Table 4:** Covariates associated with mortality. OR: odds ratio; CI: confidence interval; CTP: Child-Turcotte-Pugh; DM: diabetes mellitus; CKD: chronic kidney disease; TLC: total leukocyte count; SGOT: serum glutamic oxaloacetic transaminase; INR: international normalized ratio

Parameters	Univariate analysis			Multivariate analysis		
	OR	95% CI	P-value	OR	95% CI	P-value
Age	0.094	-0.002, 0.011	0.203	-	-	-
Sex	0.089	-0.064, 0.263	0.225	-	-	-
CURB-65 score	0.183	0.003, 0.217	0.045	0.056	0.001, 3.894	0.183
CTP score	0.412	0.011, 0.109	0.017	1.256	0.743, 2.122	0.395
DM	-0.072	-0.249, 0.075	0.282	-	-	-
CKD	-0.084	-0.470, 0.117	0.229	-	-	-
Platelet count (10^3^/µL)	0.085	-0.002, 0.005	0.348	-	-	-
TLC (10^3^/µL)	0.160	-0.004, 0.032	0.121	-	-	-
SGOT (U/L)	0.162	-0.001, 0.003	0.264	-	-	-
Creatinine (mg/dL)	-0.181	-0.086, 0.017	0.179	-	-	-
INR	0.149	-0.011, 0.060	0.177	-	-	-
Sodium (mmol/L)	0.108	-0.007, 0.020	0.316	-	-	-
Lymphocyte (>1 × 10^3^/µL)	0.202	0.162, 0.018)	0.015	0.288	0.071, 1.166	0.081
D-dimer (mg/L)	0.349	0.002, 0.027	0.021	1.374	1.004, 1.880	0.047
Ferritin (ng/mL)	-0.119	-0.004, 0.001	0.230	-	-	-

## Discussion

COVID-19 is primarily a respiratory illness caused by a coronavirus. The coronavirus spike (S) protein attaches to ACE2 receptors [10] found on the surface of many human cells, including those in the lungs and liver cholangiocytes. This allows viral entry which may be responsible for hepatic dysfunction.

Patients with cirrhosis are particularly vulnerable to infection, and cirrhosis is associated with increased mortality [11]. Several studies have demonstrated poor outcomes of COVID-19 in CLD patients during the first wave of the COVID-19 pandemic. However, studies regarding the prognosis of CWC infection during the second wave are limited. Hence, in this study, we investigated the clinical presentation and factors predicting the severity and outcome of CWC patients during the second COVID-19 wave in India. In addition, we compared CWC with CWOC as the control population.

Most of our patients presented predominantly with respiratory symptoms. Previous studies have shown that CWC patients present mainly with respiratory symptoms and have a relatively lower prevalence of gastrointestinal symptoms [12-14]. Fever and cough were predominant symptoms in this study. A multicenter cohort study by Qi et al., in China, also reported fever and cough as predominant symptoms [12]. The presentation was similar to previous studies of COVID-19 among general populations, that is, CWOC patients [15,16]. Alcoholic liver disease (ALD) was the most common etiology of cirrhosis in our study group, similar to the pre-COVID period [17,18]. However, in a study conducted by Shalimar et al. during the first wave of COVID-19 in India, the proportion of patients with CWC with ALD was less compared with their previous experience at their center [19]. A possible explanation is the non-availability of alcohol due to the lockdown imposed during the first wave. Because restriction was eased during the second wave of COVID-19, the majority of our patients had ALD. In our study, we found that AD (8%) and ACLF (10%) were the initial forms of presentation in CWC patients without any respiratory symptoms. In a study by Shalimar et al., 10/28 (35.71%) and 2/28 (7.14%) patients had AD and ACLF as presentations without respiratory symptoms [19]. Marjot et al. also reported that 21% of CWC patients who had AD had no respiratory symptoms at presentation [20]. Hence, patients with cirrhosis presenting with AD and ACLF should be evaluated for COVID-19 infection. Despite extensive evaluation, in CWC patients with ACLF, the cause of AD was not identified in 30% of patients. Hence, in this subgroup of patients, COVID-19 infection may be the cause of decompensation.

Several previous studies have shown that elderly patients and those with elevated D-dimer with multiple systemic comorbidities had severe COVID-19 infection [15,21,22]. In our study, diabetes mellitus, acute kidney injury on admission, and raised D-dimer levels were associated with severe infection. Age did not correlate with severity compared to the previous study; a possible explanation for this can be the mean age of our study population of 49 years.

In our study, elevated D-dimer, lymphocyte count, higher CURB-65, and CTP scores correlated with mortality in the CWC group. CURB-65 is a widely used severity predicting score for community-acquired pneumonia in the pre-COVID period [20]. Similar to our study, a multicenter study by Xiao et al. reported that raised D-dimer (> 1 g/L), higher CURB-65 scores, and CTP scores were associated with mortality [23]. A multinational Asian study by Sarin et al. found that high CTP score at presentation predicted mortality in the CWC group (AUROC = 0.94, HR = 19.2, 95% CI = 2.3-163.3; p < 0.001, with a sensitivity of 85.7% and specificity of 94.4%) [24]. In our study, on multivariate analysis, D-dimer of >1.1 g/L could predict mortality with a sensitivity of 96% and specificity of 52% (AUC = 0.972, p < 0.001). A case-control study by Yao et al. reported that D-dimer was the only parameter which was significantly correlated with mortality on multivariate analysis (OR = 10.17, 95% CI = 1.10-94.38; P = 0.041), and D-dimer level of >2.14 mg/L predicted in-hospital mortality with a sensitivity of 88.2% and specificity of 71.3% (AUC = 0.85, 95% CI = 0.77-0.92) [25]. Hence, raised D-dimer on admission predicts a poor prognosis.

On comparing CWC patients with the historical cohort (CWOC), overall mortality in the CWC group (50%) was double than the historical group (25%) (p = 0.003). The mortality rate in CWC patients with ACLF was 100% compared to 54% in the historical cohort (p < 0.05). Shalimar et al. reported a mortality rate of 42.3% (11/26) in the CWC group compared to 23.1% (18/78) in the historical cohort (p = 0.077). In the same study, all CWC patients with ACLF (9/9) died compared to 53.3% (16/30) in the historical cohort (p = 0.015) [19]. However, Bajaj et al. did not report any difference in mortality in the CWC ACLF group and the CWOC ACLF group (n = 6, 55% vs n = 10, 36%; p = 0.25) [14]. The direct viral effect, inflammation induced by SARS-CoV-2, liver ischemia-reperfusion injury, previous liver disease aggravation, and medical treatment could be contributing factors for the high mortality in this group of patients. Most patients in our study succumbed due to respiratory rather than gastrointestinal complications. Previous registries have shown that CWC patients have a poor prognosis with a mortality rate of 13-42.3%, which is mostly related to pulmonary complications followed by liver-related causes [14,19,20,26,27]. A large case-control study on the US population by Wang et al. reported an increased risk of infection, hospitalization, and mortality among COVID-19 patients with CLD compared to COVID-19 patients without CLD [28]. However, in our study, we did not compare patients between these two groups. Table 5 lists the studies reported during the first wave of the COVID-19 pandemic predicting the outcomes of CWC patients.

**Table 5:** Studies during the first wave of the COVID-19 pandemic predicting the outcome of CWC patients. CWC: cirrhosis with COVID-19; COVID-19: coronavirus disease 2019; CCI: Charlson Comorbidity Index; ACLF: acute on chronic liver failure; CLD: chronic liver disease; SARS-CoV-2: severe acute respiratory syndrome coronavirus 2; AST: aspartate transaminase; ALT: alanine transaminase; OR: odds ratio; CI: confidence interval; HR: hazard ratio

Authors	Study population	Major findings
Bajaj et al. [14]	37 patients with cirrhosis plus COVID-19 were matched with 108 patients with COVID-19 and 127 patients with cirrhosis	Patients with cirrhosis plus COVID-19 had higher mortality compared with patients with COVID-19 (30% vs. 13%, p = 0.03) but not between patients with cirrhosis plus COVID-19 and patients with cirrhosis (30% vs. 20%, p = 0.16). In the entire group, CCI (OR = 1.23, 95% CI = 1.11-1.37; p < 0.0001) was the only variable predictive of mortality on multivariable regression
Shalimar et al. [19]	28 COVID-19 patients with cirrhosis	The mortality rate in COVID-19 patients was 42.3% (11/26) compared to 23.1% (18/78) in the historical controls (p = 0.077). All COVID-19 patients with ACLF (9/9) died compared to 53.3% (16/30) in ACLF patients in the historical control group (p = 0.015). Requirement of mechanical ventilation independently predicted mortality (HR = 13.68)
Marjot et al. [20]	745 patients with CLD and SARS-CoV-2 (including 386 with and 359 without cirrhosis)	Mortality was 32% in patients with cirrhosis compared to 8% in those without (p < 0.001). Factors associated with death in the total CLD cohort were age (OR = 1.02; 1.01–1.04), Child-Pugh A (OR = 1.90; 1.03–3.52), B (OR = 4.14; 2.4–7.65), or C (OR = 9.32; 4.80–18.08) cirrhosis, and alcohol-related liver disease (OR = 1.79; 1.03–3.13)
Sarin et al. [24]	228 patients (185 CLD without cirrhosis and 43 with cirrhosis)	Liver-related complications increased (p < 0.05) with stage of liver disease. CTP score of 9 or more at presentation predicted high mortality. Rising bilirubin and AST/ALT ratio predicted mortality among cirrhosis patients
Kim et al. [26]	867 patients with CLD plus COVID-19	The overall all-cause mortality was 14.0% (n = 121), and 61.7% (n = 535) had severe COVID-19. Liver-specific factors associated with independent risk of higher overall mortality included ALD (HR = 2.42, 95% CI = 1.29–4.55), decompensated cirrhosis (HR = 2.91, 95% CI = 1.70–5.00), and HCC (HR = 3.31, 95% CI = 1.53–7.16]. Other factors included increasing age, diabetes, hypertension, chronic obstructive pulmonary disease, and current smoker
Wang et al. [28]	1,034,270 patients with CLD, 16,530 with COVID-19, and 820 with both COVID-19 and CLD	Patients with CLD were at a significantly increased risk for COVID-19 compared with patients without CLD. African Americans with CLD were twice more likely to develop COVID-19 than Caucasians. Patients with COVID-19 and a recent encounter of CLD had a death rate of 10.3% versus 5.5% among COVID-19 patients without CLD (p < 0.001) and a hospitalization rate of 41.0% versus 23.9% among COVID-19 patients without CLD (p < 0.001)

Study limitations

A major limitation of our study was that it was a retrospective study with a small sample size. Being a tertiary care center for COVID-19, selection bias toward more severe cases cannot be excluded. Moreover, we did not compare patients of CWC with patients of CWOC. Results of our study need to be validated by prospective studies with a larger sample size before generalization.

## Conclusions

The presence of cirrhosis in COVID-19 patients was associated with a poor outcome. The occurrence of ACLF in COVID-19 is associated with 100% mortality. Serum D-dimer level not only predicts severity but also mortality in CWC patients. CURB-65 score on admission is a simple factor that can be used to determine the outcome in these patients.
